# Impact of rapid rehabilitation surgery on perioperative nursing in patients undergoing cardiac surgery: A meta‐analysis

**DOI:** 10.1111/jocs.17226

**Published:** 2022-11-30

**Authors:** Wenjuan Feng, Jing Zhou, Yu Lei, Wenmin Chen, Yongpin Miao, Xintong Fu, Jinghong Pi, Min Zhang, Zhuhui Na, Wenrong Lou

**Affiliations:** ^1^ Cardiac Surgery Department Yan'an Hospital Kunming China; ^2^ Department of Stomatology Kunming Yanan Hospital Kunming China; ^3^ Key Laboratory of Tumor Immunological Prevention and Treatment of Yunnan Province Yan'an Hospital Kunming Yunnan China; ^4^ E.N.T. Department Yan'an Hospital Kunming Yunnan China

**Keywords:** cardiac surgery, enhanced recovery after surgery, meta‐analysis, perioperative period, randomized controlled trial

## Abstract

**Objective:**

To systematically evaluate the effect of enhanced recovery after surgery (ERAS) on perioperative nursing of patients undergoing cardiac surgery.

**Methods:**

A systematic literature search was performed in PubMed, Embase, Web of science, Cochrane, CNKI, Wanfang, and VIP using predefined search strings from inception of database to May 2021. Randomized control trials (RCTs) with sample size >40 on cardiac surgery with either ERAS nursing or routine nursing reporting extubation (trachea) time, length of stay, out of bed activity time, and nursing satisfaction were included in the analysis. Stata SE 12.0 software was used for statistical analysis.

**Results:**

A total of 27 RCTs were included. All the included studies were Chinese due to lack of studies in English. The results of meta‐analysis showed that the extubation time standardized mean difference ([SMD] = −3.11; 95% confidence interval [CI]: −3.77, −2.45; *p* < .001), out of bed activity time (SMD = −2.89; 95% CI: −3.34, −2.44; *p* < .001), and hospitalization time (SMD = −2.08; 95% CI: −2.37, −1.79; *p* < .001) of cardiac surgery patients with ERAS nursing was significantly shorter than those with routine nursing. The patient's satisfaction after surgery with ERAS was higher than that of routine nursing relative risk ([RR] = 1.24; 95% CI: 1.18, 1.30; *p* < .001).

**Conclusion:**

ERAS nursing can accelerate perioperative rehabilitation of patients undergoing cardiac surgery and highly accepted by patients.

## 1 INTRODUCTION

The concept of enhanced recovery after surgery (ERAS) was first proposed by Professor Henrik Kehlet of the University of Copenhagen in Denmark, also known as fast‐track surgery (FTS).^1^ ERAS is a multimodal and multidisciplinary evidence‐based surgical nursing method, which aims to optimize perioperative management and prognosis, so as to reduce patients’ surgical stress response, reduce postoperative complications, promote functional recovery, shorten length of hospital stay, and achieve rapid rehabilitation.^2^ ERAS was first applied to patients undergoing colorectal surgery.^3^ At present, it has been widely used all over the world. Different ERAS guidelines or consensus have been published in many fields, such as rectal/pelvic surgery,^4^ pancreaticoduodenectomy,^5^ radical cystectomy,^6^ gastrectomy,^7^ pulmonary surgery,^8^ colorectal surgery,^9^ gynecology/oncology,^10^ cesarean section,^11^ and cardiac surgery.^12^ Most studies have shown that using ERAS nursing can reduce patients' total length of stay and hospitalization expenses of patients, and improve the quality of life and satisfaction by reducing insulin resistance and inflammatory reaction caused by surgery.^13^, ^14^, ^15^

According to the global disease burden report, cardiovascular diseases are the leading cause of disease burden in the world including China.^16^, ^17^, ^18^ Cardiac surgery is widely used as an effective treatment to reduce the mortality of cardiovascular patients.^19^ Although ERAS is still a relatively new concept in the field of cardiac surgery it is expected to play an important role in the perioperative nursing of cardiac surgery. In 2019, guidelines on ERAS in the perioperative period of cardiac surgery were published.^12^

Recently, the safety and effectiveness of ERAS in cardiac surgery patients has been increasing studied. A recent study reported a significant reduction in intensive care time, postoperative stay, and length of hospital stay of cardiac surgery patients with ERAS nursing compared with traditional nursing.^20^ In the past, many systematic reviews and meta‐analyses have reported the effectiveness and safety of ERAS nursing in different surgical operations.^21^, ^22^, ^23^ However, no meta‐analysis on ERAS nursing in cardiac surgery has been available. Therefore, this study focuses to study the effect of ERAS on perioperative nursing of cardiac surgery patients, to provide evidence on the feasibility, safety, and reliability of ERAS nursing in cardiac surgery patients.

## 2 METHODS

### 2.1 Study design

This systematic review and meta‐analysis was conducted to evaluate the effect of ERAS on perioperative nursing of patients undergoing cardiac surgery as per the “Preferred Reporting Items for Systemic reviews and Meta‐analyses” guidelines.^24^ An extensive literature search was performed in various databases such as PubMed, Embase, Web of Science, and Cochrane Central Register of Controlled Trials to identify relevant English articles, while CNKI, VIP, and Wanfang for Chinese articles. Keywords used for literature search include, enhanced recovery after surgery, ERAS, fast track surgery, FTS, enhanced recovery after surgery, cardiac surgery, periodic period of cardiac surgery, periodic nursing, nursing, and nursing care. All articles published from inception to May 2021 were considered.

### 2.2 Outcomes

Extubation (trachea) time, length of hospital stay, out of bed activity time, and nursing satisfaction of cardiac surgery patients with ERAS nursing or routine nursing were considered as outcomes of this analysis.

### 2.3 Inclusion and exclusion criteria

All randomized controlled trials comparing ERAS nursing with routine nursing in patients undergoing cardiac surgery with a sample size >40 reporting any one of the outcomes considered for the study were included in the analysis.

Single‐arm studies, nonrandomized controlled studies, meta‐analysis, systematic literature reviews, narrative reviews, case reports, conference proceedings, one or two types of cardiac surgery (like replacing two valves or replacing one valve and plastic surgery or valve replacement and radiofrequency ablation), and studies with a sample size of <40 were excluded.

### 2.4 Screening and eligibility assessment

After removing duplicates, all the studies were screened as per the inclusion criteria by two independent reviewers to ensure that the studies met prespecified study inclusion criteria. Any disagreement was resolved by the third reviewer.

### 2.5 Data extraction

Data from included studies regarding author, year of publication, title, study design, demographics of the study population, and outcomes of interest was extracted by two independent reviewers, that are trained and certified on meta‐analysis from West China Hospital, into standardized MS Office Excel.

### 2.6 Assessment of risk bias

Two researchers independently evaluated the bias risk of the included studies and cross checked the results. The bias risk of randomized control trials (RCTs) was evaluated using the RCT bias risk assessment tool recommended in Cochrane manual 5.1.0.^25^

### 2.7 Statistical analysis

STATA 12.0 software was used for analysis. The standardized mean difference (SMD) was used for effect analysis for the continuous variables and the relative risk (RR) with 95% confidence interval (CI) was used as the effect analysis for the binary variables. The heterogeneity between the included study results was analyzed by *χ* ^2^ test (the test level was *α* = .1) and *I* ^2^ to quantitatively judge the heterogeneity. If there is no statistical heterogeneity among the study results, the fixed effect model is used for meta‐analysis; if there is statistical heterogeneity among the study results, the source of heterogeneity is further explored by meta‐regression and sensitivity analysis. The publication bias of the included literature is evaluated by the combination of Begg's and Egger's test and funnel plot. If the *p* value for heterogeneity is <.05 or *I* ^2^ is ≥50% was considered as statistically significant.

## 3 RESULTS

### 3.1 Study selection

**Figure 1:**
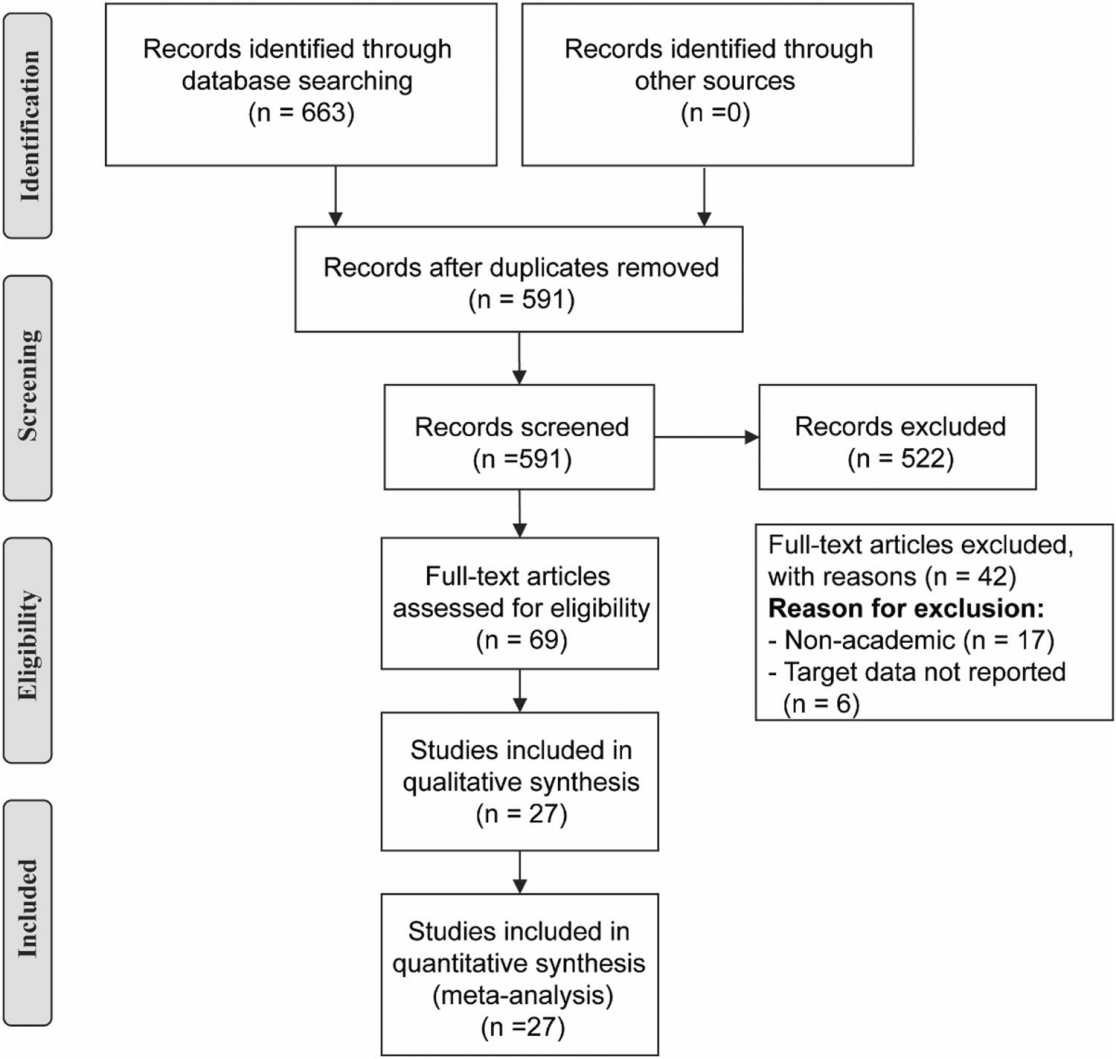
Preferred Reporting Items for Systemic reviews and Meta‐Analyses flow chart

A total of 663 articles (PubMed: 103, Web of Science: 40, Embase: 97, Cochrane: 65, CNKI: 219, Wanfang: 94, VIP: 45) were retrieved initially from all databases. After removal of duplicates and screening for inclusion/exclusion criteria, a total of 27 RCTs were finally included in the analysis. The literature screening process and results are shown in Figure 1.

### 3.2 Study characteristics

Overall, 2455 patients (intervention: 1232 and control: 1223 patients) were included from 27 studies (English: 0 and Chinese: 27) published from 2016 to 2021. The basic characteristics of the included studies are shown in Table 1.

**Table 1:** Basic characteristics of the included studies

Study	Patient (children/adult)	Sample size	Age (years or months)	Outcome indicators
		T/C	T/C	
Cang et al.^26^	Children	40/40	5.45 ± 2.02/5.62 ± 1.92	Extubation time, length of hospital stay, nursing satisfaction
Chen^27^	Adult	49/49	56.55 ± 2.38/55.37 ± 2.74	Extubation time, length of hospital stay, out of bed activity time
Ding^28^	Adult	24/23	56.50 ± 3.33/56.41 ± 3.26	Extubation time
Gao^29^	Adult	84/84	33.56 ± 10.87/34.28 ± 10.61	Nursing satisfaction
Guo^30^	Children	36/36	7.3 ± 2.8/7.5 ± 2.6	Extubation time, length of hospital stay, nursing satisfaction
He and Zhou^31^	Adult	60/60	41.58 ± 3.47/41.39 ± 3.55	Out of bed activity time
Hu^32^	Adult	40/40	57 ± 1.71/55 ± 1.65	Extubation time, length of hospital stay, out of bed activity time
Huang^33^	Adult	33/33	59 ± 16/58 ± 16	Extubation time, length of hospital stay
Jiang^34^	Adult	56/56	57.3 ± 2.9/57.4 ± 2.2	Length of hospital stay, out of bed activity time, nursing satisfaction
Liu^35^	Children	25/25	9.34 ± 0.75/9.32 ± 0.77	Extubation time, length of hospital stay, out of bed activity time
Liu^36^	Adult	23/23	41.54 ± 1.35/41.67 ± 1.24	Extubation time, length of hospital stay
Ma et al.^37^	Adult	40/40	52.6 ± 4.3/52.8 ± 4.1	Extubation time, length of hospital stay, out of bed activity time
Mao^38^	Children	100/100	8.54 ± 2.61a/8.32 ± 2.23a	Length of hospital stay
Peng^39^	Adult	41/41	58.2 ± 7.4/55.6 ± 6.5	Extubation time, length of hospital stay, out of bed activity time, nursing satisfaction
Qiu^40^	Adult	49/49	40.64 ± 5.29/39.54 ± 6.24	Extubation time, length of hospital stay, out of bed activity time
Ruan^41^	Adult	21/21	41.47 ± 2.27	Extubation time, length of hospital stay, out of bed activity time
Shu and Li^42^	Adult	41/41	66 ± 6/65 ± 7	Nursing satisfaction
Tang^43^	Adult	42/42	51 ± 3/48 ± 4	Extubation time, length of hospital stay, out of bed activity time, nursing satisfaction
Wang^44^	Adult	40/40	56.23 ± 4.52	Extubation time, nursing satisfaction
Wang^45^	Adult	58/58	47.54 ± 6.31/48.25 ± 8.53	Extubation time, length of hospital stay, out of bed activity time
Wang^46^	Adult	51/51	43.8 ± 8.2/42.4 ± 8.6	Length of hospital stay, out of bed activity time
Wen and Zhou^47^	Adult	33/33	47.5 ± 2.4/49.0 ± 2.6	Extubation time, length of hospital stay, out of bed activity time
Xie^48^	Adult	39/39	58.7 ± 5.4/58.3 ± 5.1	Length of hospital stay, out of bed activity time, nursing satisfaction
Yang et al.^49^	Children	43/43	6.4 ± 1.1/6.6 ± 1.3	Extubation time, length of hospital stay, nursing satisfaction
Zhang^50^	Adult	49/41	41.53 ± 10.31/41.98 ± 10.37	Extubation time, length of hospital stay, nursing satisfaction
Zhao^51^	–	70/70	–	Extubation time, length of hospital stay
Zhong^52^	Children	45/45	5.7 ± 2.3/6.1 ± 2.5	Extubation time, length of hospital stay

### 3.3 Outcomes

#### 3.3.1 Extubation time

**Figure 2:**
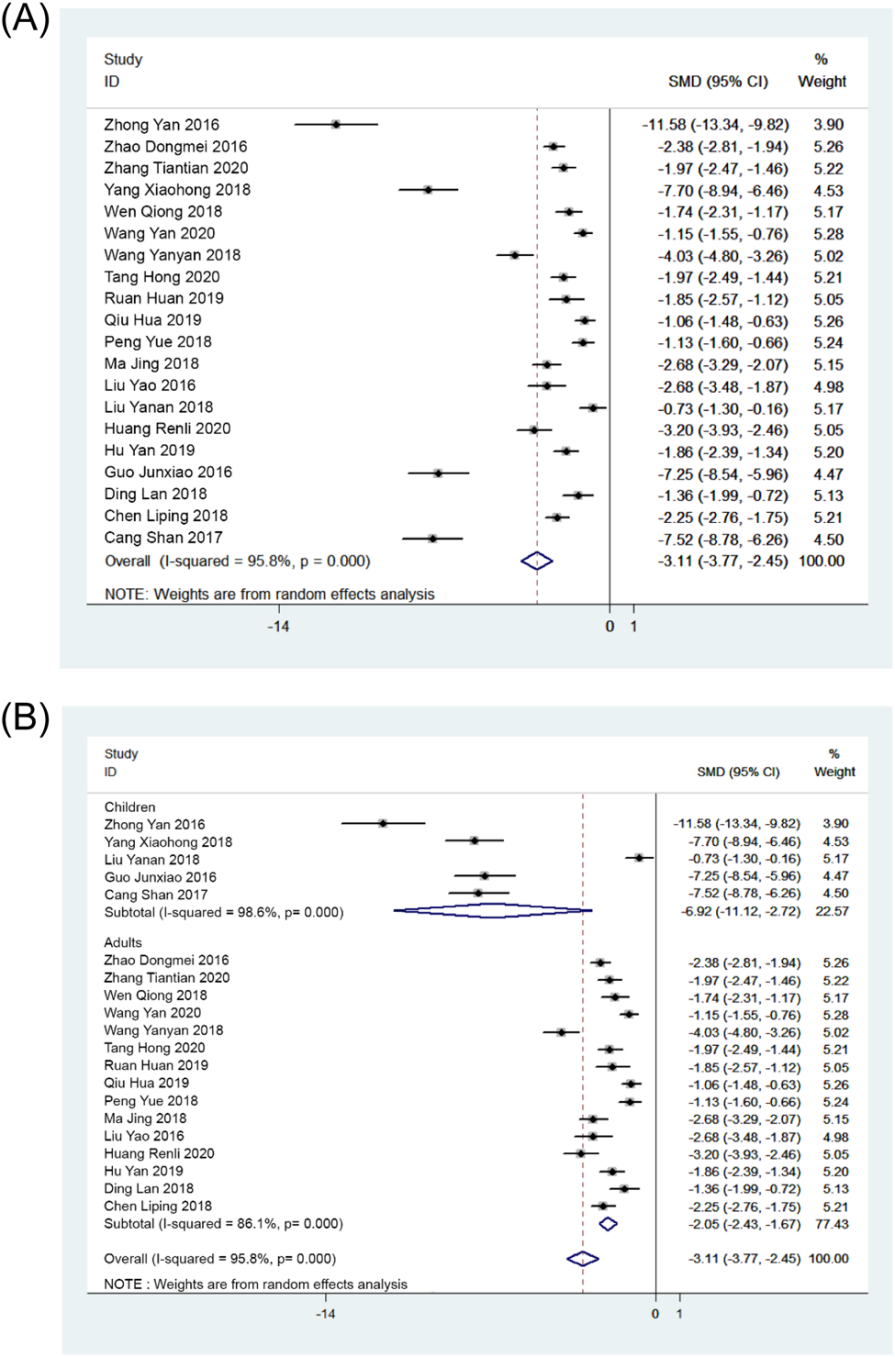
Forest plot. (A) Extubation time. (B) Subgroup analysis of extubation time. CI, confidence interval; SMD, standardized mean differences.

Of 27 studies, 20 studies reported extubation time. The combined SMD value was −3.11 (95% CI: −3.77, −2.45; *Z* = 9.2; Figure 2A) with *p* < .001 indicating that the extubation time of patients with ERAS nursing was significantly lower than that of patients with routine nursing. From subgroup analysis, the SMD value for children was −6.92 (95% CI: −11.21, −2.72) and for adults was −3.11 (95% CI: −3.77, −2.45; Figure 2B), and the extubation time of children and adults was significantly shorter with ERAS nursing than that of routine nursing (children: *Z* = 3.23, *p* = .001; adults: *Z* = 10.65, *p* < .001). A significant heterogeneity was observed among the included studies (*I* ^2^ = 95.8%, *p* < .001).

#### 3.3.2 Length of hospital stay

**Figure 3:**
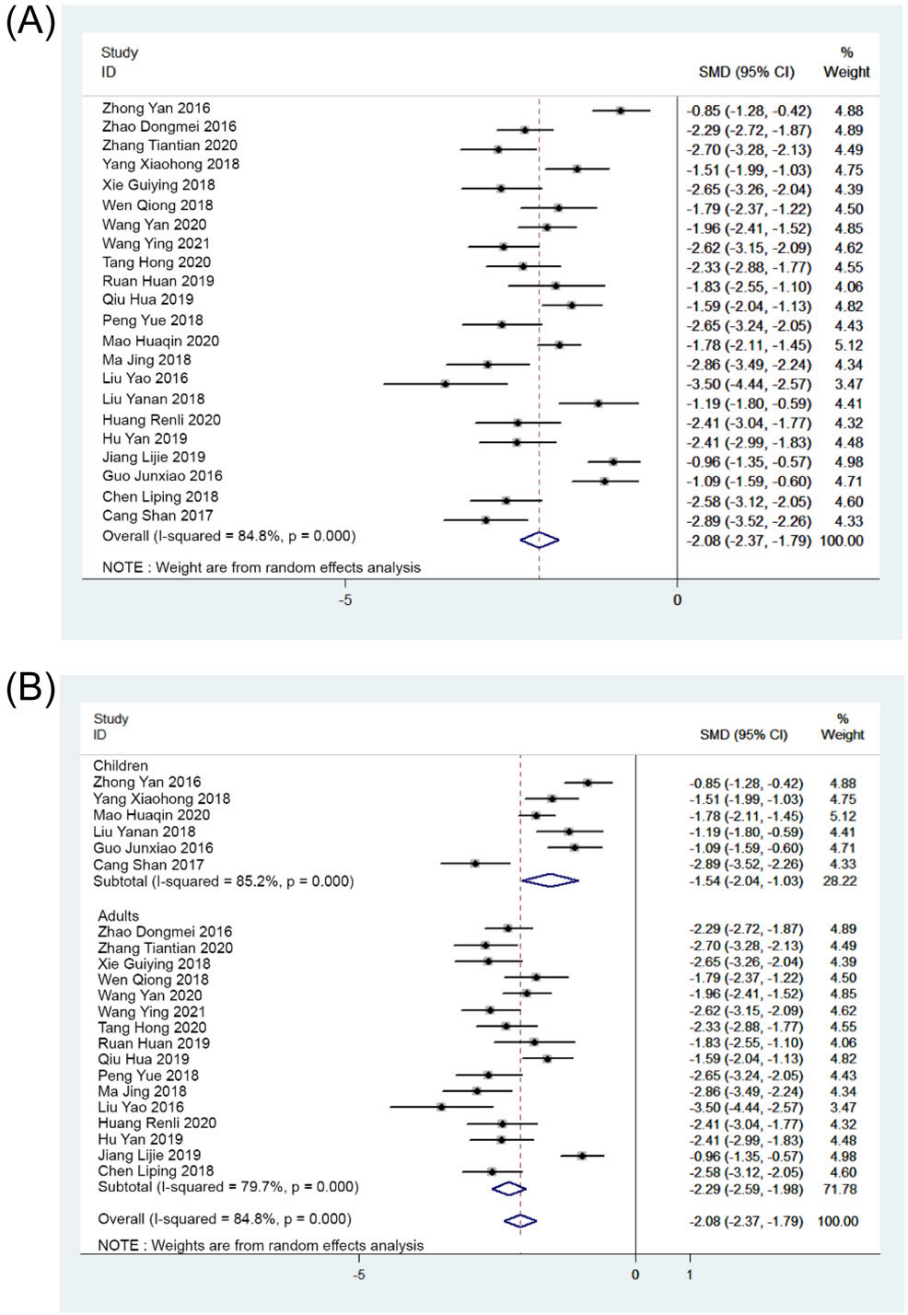
Forest plot. (A) Length of hospital stay. (B) Subgroup analysis of length of hospital stay. CI, confidence interval; SMD, standardized mean differences.

A total of 20 studies reported length of hospital stay. A significant heterogeneity was observed among studies (*I* ^2^ = 84.8%, *p* < .001), random effect model was used for analysis. The combined SMD value was −2.08 (95% CI: −2.37, −1.79, Figure 3A) with *p* < .001 (*Z* = 14.22) indicating that the length of hospital stay of patients with ERAS nursing was significantly lower than that of patients with routine nursing. Subgroup analysis showed that with ERAS nursing, the length of hospital stay of both children and adults was significantly shorter than that of routine nursing (children: *Z* = 5.97, *p* < .001; adults: *Z* = 14.67, *p* < .001; Figure 3B).

#### 3.3.3 Out of bed activity time

**Figure 4:**
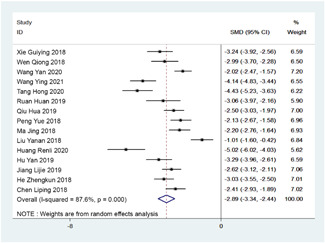
Forest plot of out of bed activity time. CI, confidence interval; SMD, standardized mean differences.

Out of bed activity was reported by 15 studies, which were included in the analysis. Out of bed activity time of patients on ERAS nursing was significantly lower than that of patients on routine nursing as analyzed by random effect model (*I* ^2^ = 87.6%, *p* < .001) with SMD of −2.89; 95% CI: −3.34, −2.44; *p* < .001 (*Z* = 12.63; Figure 4).

#### 3.3.4 Nursing satisfaction

**Figure 5:**
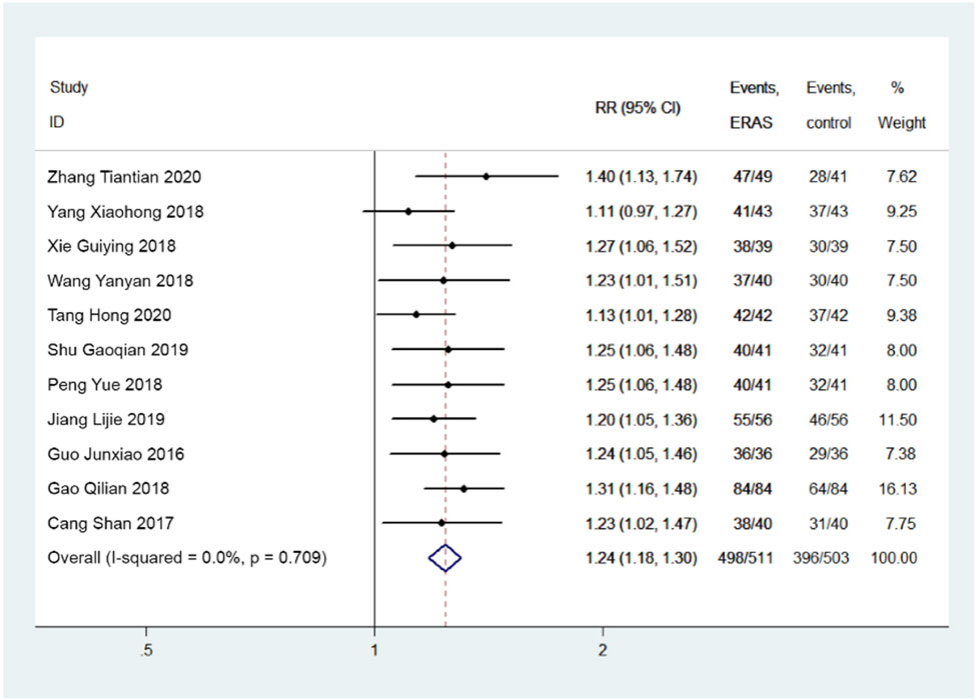
Forest plot of out of bed activity time. CI, confidence interval; ERAS, enhanced recovery after surgery.

A total of 11 studies that reported nursing satisfaction were included in the analysis. No heterogeneity was observed among studies (*I* ^2^ = 0%, *p* = .709) hence fixed effect model was used for analysis. Patients on ERAS nursing showed 1.24 times higher satisfaction compared to routine nursing as analyzed from fixed effect model (*I* ^2^ = 0%; *p* = .709) with RR of 1.24; 95%  CI: 1.18–1.30; *p* < .001 (*Z* = 8.73; Figure 5).

### 3.4 Publication bias

**Figure 6:**
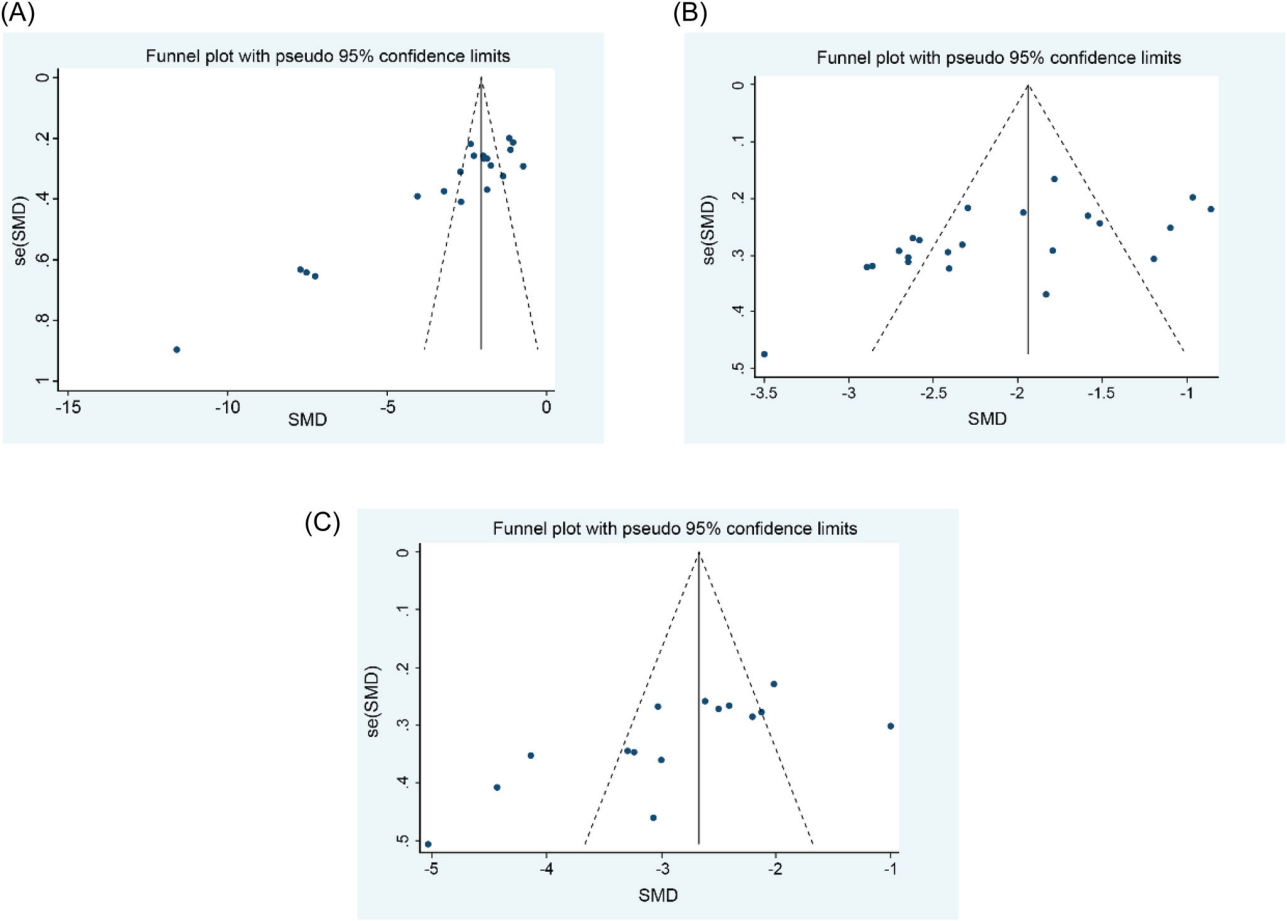
Funnel plots. (A) Extubation time.  (B) Length of hospital stay. (C) Out of bed activity time. SMD, standardized mean differences.

The results of Begg's and Egger's tests showed that there was publication bias among the studies included reporting extubation time, length of hospital stay, and out of bed activity time (*p* < .05; Table 2; Figure 6), and the funnel plots showed asymmetric distribution, while no publication bias was observed among the studies reporting nursing satisfaction (*p* > .05). The results showed that there was little difference between the effect values before extubation (effect value: −3.108) and after extubation (effect value: −3.579) (*p* < .001), but there was no change in the effect values before and after hospitalization and out of bed activity time (*p* < .001). Hence, it can be considered that the existence of publication bias had no effect on the results of meta‐analysis.

**Table 2:** Publication bias test results

Outcome indicators	Begg's test	Egger's test
Extubation time	<0.001	<0.001
Length of hospital stay	0.009	0.005
Out of bed activity time	0.023	0.005
Nursing satisfaction	0.186	0.141

### 3.5 Source of heterogeneity

**Figure 7:**
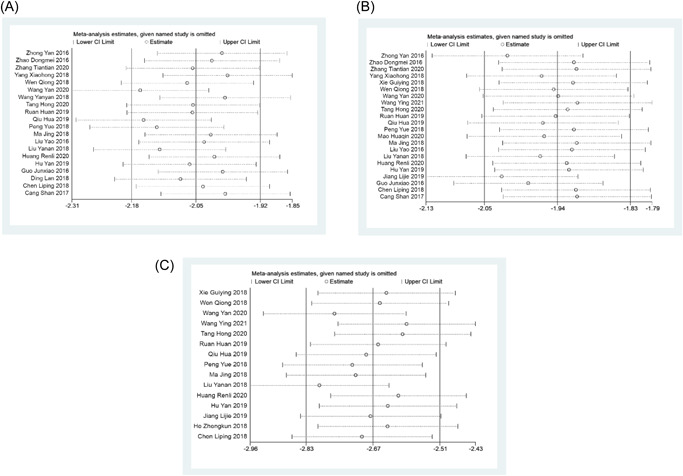
Sensitivity analysis. (A) Extubation time. (B) Length of hospital stay. (c) Out of bed activity time. CI, confidence interval.

A significant heterogeneity was observed among the studies reporting extubation time, length of hospital stay, and out of bed activity time. The meta‐regression analysis showed that the year of publication and the population (children or adults) may be the source of heterogeneity in extubation time and out of bed activity time, while the population (children or adults) may be the source of heterogeneity in length of hospital stay (Table 3). The results of sensitivity analysis showed that the outcome index is extubation time. The sensitivity and robustness between the studies reporting length of hospital stay and out of bed activity time were high and poor respectively, which may be the source of heterogeneity, as shown in Figure 7.

**Table 3:** Univariate meta‐regression analysis of extubation time, length of stay, and out of bed activity time

Variable	*β*	SE	95% CI	*t* value	*p* Value
Extubation time					
Year of publication	1.08	0.41	0.214–1.954	2.62	.017
Constant	−2191.3	835.71	−3947.06 to −435.54	−2.62	.017
Population	4.678	1.03	2.52–6.84	4.55	<.001
Constant	−11.44	1.87	−15.36 to −7.52	−6.13	<.001
Length of hospital stay					
Year of publication	−.06	0.10	−0.27 to 0.16	−0.54	.593
Constant	111.42	209.12	−324.80 to 547.64	0.53	.600
Population	−.75	0.29	−1.36 to −0.14	−2.56	.019
Constant	−.79	0.52	−1.87 to 0.30	−1.51	.147
Out of bed activity time					
Year of publication	−.60	0.23	−1.083 to 0.10	−2.61	.022
Constant	1193.35	458.64	202.52–2184.18	2.60	.022
Population	−2.02	0.90	−3.955 to −0.08	−2.25	.042
Constant	1.013	1.75	−2.76 to 4.80	0.58	.572

Abbreviations: CI, confidence interval; SE, standard error.

### 3.6 Assessment of risk bias of studies included

The quality of the included studies was found to be good, and the risk of bias results are presented in Table 4.

**Table 4:** Bias risk assessment of the included studies

Included study	Randomization	Blind method	Assign hide	Integrity of result data	Selective reporting of study results	Other sources of bias
Cang^26^	Random number table method	Not reported	Not reported	complete	No	Unclear
Chen^27^	Random number table method	Not reported	Not reported	Complete	No	Unclear
Ding^28^	Random, unreported	Not reported	Not reported	Complete	No	Unclear
Gao^29^	Random, unreported	Not reported	Not reported	Complete	No	Unclear
Guo^30^	Random number table method	Not reported	Not reported	Complete	No	Unclear
He^31^	Lottery method	Not reported	Not reported	Complete	No	Unclear
Hu^32^	Random, unreported	Not reported	Not reported	Complete	No	Unclear
Huang^33^	Random number table method	Not reported	Not reported	Complete	No	Unclear
Jiang^34^	Random, unreported	Not reported	Not reported	Complete	No	Unclear
Liu^35^	Random number table method	Not reported	Not reported	Complete	No	Unclear
Liu^36^	Random, unreported	Not reported	Not reported	Complete	No	Unclear
Ma^37^	Random number table method	Not reported	Not reported	Complete	No	Unclear
Mao^38^	Random number table method	Not reported	Not reported	Complete	No	Unclear
Peng^39^	Random, unreported	Not reported	Not reported	Complete	No	Unclear
Qiu^40^	Random lottery	Not reported	Not reported	Complete	No	Unclear
Ruan^41^	Random number table method	Not reported	Not reported	Complete	No	Unclear
Shu^42^	Random, unreported	Not reported	Not reported	Complete	No	Unclear
Tang^43^	Random number table method	Not reported	Not reported	Complete	No	Unclear
Wang^44^	Random distribution table method	Not reported	Not reported	Complete	No	Unclear
Wang^45^	Random number table method	Not reported	Not reported	Complete	No	Unclear
Wang^46^	Random, unreported	Not reported	Not reported	Complete	No	Unclear
Wen^47^	Random lottery	Not reported	Not reported	Complete	No	Unclear
Xie^48^	Random number table method	Not reported	Not reported	Complete	No	Unclear
Yang^49^	Random, unreported	Not reported	Not reported	Complete	No	Unclear
Zhang^50^	Random, unreported	Not reported	Not reported	Complete	No	Unclear
Zhao^51^	Random, unreported	Not reported	Not reported	Complete	No	Unclear
Zhong^52^	Random, unreported	Not reported	Not reported	Complete	No	Unclear

## 4 DISCUSSION

ERAS nursing involves, preoperative (to optimize patient before surgery), intraoperative and postoperative care (to enhance patient rehabilitation and recovery). Preoperative ERAS includes preadmission counseling, nutritional screening/support, medical optimization of chronic diseases, selective use of bowel preparation, avoid prolonged fasting, carbohydrate loading, antibiotic, and thromboprophylaxis if necessary. Minimally invasive surgery techniques, standardized anesthesia techniques, selective use of drains, avoiding fluid overload, and maintaining normal body temperature are taken care in intraoperative ERAS. Postoperative ERAS includes avoidance of nasogastric tubes, early oral intake of liquids and solids, removing urinary catheter and intravenous infusion tube as early as possible, preventing nausea and vomiting, use of nonopioid analgesics, early mobilization, and preparation for early discharge.^53^, ^54^

It is speculated that the combination of one or more of the above measures may be applied to patients undergoing cardiac surgery to accelerate their rehabilitation and make them physically and mentally comfortable during the nursing process, so as to shorten the extubation time, out of bed activity time and length of stay and improve their satisfaction with nursing. Li et al.^55^ reported shorter discharge time and intensive care unit treatment time after the implementation of ERAS nursing in patients undergoing cardiac valve surgery compared with routine nursing. A recent study on patients undergoing minimally invasive aortic valve or mitral valve surgery reported significantly shorter length of hospital stay and lower hospitalization cost with ERAS nursing compared with patients on routine nursing.^56^ A study on Chinese patients showed that compared with routine nursing, the extubation time and out of bed activity time of patients undergoing cardiac surgery was significantly shorter in those receiving ERAS nursing and significantly improved nursing satisfaction.^35^ In consistent with the reported studies, the results of the present meta‐analysis also showed significantly shorter extubation time, out of bed activity time and length of hospital stay of cardiac surgery patients with ERAS nursing compared to routine nursing, while the nursing satisfaction of ERAS was higher than that of routine nursing, indicating that ERAS nursing accelerates surgical recovery, improves patient outcomes and highly acceptable by patients.

In the present meta‐analysis, although meta‐regression showed that the publication year and the population (children or adults) of the studies may be the sources of heterogeneity, it can only explain part of the sources of heterogeneity. As all studies included are Chinese and the type of cardiac surgery was not specified, the type and numbers of cardiac surgical procedures undergone by patients may be different in the included studies, which may be the reason of heterogeneity among the studies. However, because ERAS is not widely used in the field of cardiac surgery and only a few studies have been published, hence conducting meta‐analysis on ERAS nursing in each cardiac surgery was challenging.

The present meta‐analysis has certain limitations: All the studies included in the analysis are in Chinese as there is lack of studies in English; due to the lack of relevant literature, subgroup analysis of each cardiac surgery was not conducted; the source of heterogeneity has not been explored clearly, which needs to be further explored through subgroup analysis on the type of cardiac surgery.

## AUTHOR CONTRIBUTIONS

All authors approved final version of the manuscript.

## CONFLICT OF INTEREST

The authors declare no conflict of interest.

## References

[1] Kehlet H . Multimodal approach to control postoperative pathophysiology and rehabilitation. Br J Anaesth. 1997;78(5):606‐617. 9175983 10.1093/bja/78.5.606

[2] Kehlet H , Wilmore DW . Evidence‐based surgical care and the evolution of fast‐track surgery. Ann Surg. 2008;248(2):189‐198. 18650627 10.1097/SLA.0b013e31817f2c1a

[3] Fearon KCH , Ljungqvist O , Von Meyenfeldt M , et al. Enhanced recovery after surgery: a consensus review of clinical care for patients undergoing colonic resection. Clin Nutr. 2005;24(3):466‐477. 15896435 10.1016/j.clnu.2005.02.002

[4] Nygren J , Thacker J , Carli F , et al. Guidelines for perioperative care in elective rectal/pelvic surgery: enhanced recovery after surgery (ERAS®) Society recommendations. Clin Nutr. 2012;31(6):801‐816. 23062720 10.1016/j.clnu.2012.08.012

[5] Lassen K , Coolsen MME , Slim K , et al. Guidelines for perioperative care for pancreaticoduodenectomy: enhanced recovery after surgery (ERAS®) Society recommendations. Clin Nutr. 2012;31(6):817‐830. 23079762 10.1016/j.clnu.2012.08.011

[6] Cerantola Y , Valerio M , Persson B , et al. Guidelines for perioperative care after radical cystectomy for bladder cancer: enhanced recovery after surgery (ERAS®) Society recommendations. Clin Nutr. 2013;32(6):879‐887. 24189391 10.1016/j.clnu.2013.09.014

[7] Mortensen K , Nilsson M , Slim K , et al. Consensus guidelines for enhanced recovery after gastrectomy. Br J Surg. 2014;101(10):1209‐1229. 25047143 10.1002/bjs.9582

[8] Batchelor TJP , Rasburn NJ , Abdelnour‐Berchtold E , et al. Guidelines for enhanced recovery after lung surgery: recommendations of the enhanced recovery after surgery (ERAS®) Society and the European Society of Thoracic Surgeons (ESTS). Eur J Cardiothorac Surg. 2019;55(1):91‐115. 30304509 10.1093/ejcts/ezy301

[9] Gustafsson UO , Scott MJ , Hubner M , et al. Guidelines for perioperative care in elective colorectal surgery: enhanced recovery after surgery (ERAS®) Society recommendations: 2018. World J Surg. 2019;43(3):659‐695. 30426190 10.1007/s00268-018-4844-y

[10] Nelson G , Bakkum‐Gamez J , Kalogera E , et al. Guidelines for perioperative care in gynecologic/oncology: enhanced recovery after surgery (ERAS) Society recommendations‐2019 update. Int J Gynecol Cancer. 2019;29(4):651‐668. 30877144 10.1136/ijgc-2019-000356

[11] Macones GA , Caughey AB , Wood SL , et al. Guidelines for postoperative care in cesarean delivery: enhanced recovery after surgery (ERAS) Society recommendations (part 3). Am J Obstet Gynecol. 2019;221(3):247.e1‐247.e9. 10.1016/j.ajog.2019.04.012 30995461

[12] Engelman DT , Ben Ali W , Williams JB , et al. Guidelines for perioperative care in cardiac surgery: enhanced recovery after surgery Society recommendations. JAMA Surg. 2019;154(8):755‐766. 31054241 10.1001/jamasurg.2019.1153

[13] Pang KH , Groves R , Venugopal S , Noon AP , Catto JWF . Prospective implementation of enhanced recovery after surgery protocols to radical cystectomy. Eur Urol. 2018;73(3):363‐371. 28801130 10.1016/j.eururo.2017.07.031

[14] Afonso A , Oskar S , Tan KS , et al. Is enhanced recovery the new standard of care in microsurgical breast reconstruction? Plast Reconstr Surg. 2017;139(5):1053‐1061. 28092334 10.1097/PRS.0000000000003235 PMC5640259

[15] Rao JH , Zhang F , Lu H , et al. Effects of multimodal fast‐track surgery on liver transplantation outcomes. Hepatobiliary Pancreat Dis Int. 2017;16(4):364‐369. 28823365 10.1016/S1499-3872(17)60020-1

[16] Roth GA , Abate D , Abate KH , et al. Global, regional, and national age‐sex‐specific mortality for 282 causes of death in 195 countries and territories, 1980–2017: a systematic analysis for the Global Burden of Disease Study 2017. Lancet. 2018;392(10159):1736‐1788. 30496103 10.1016/S0140-6736(18)32203-7 PMC6227606

[17] GBD Risk Factor C , et al. Global, regional, and national comparative risk assessment of 84 behavioural, environmental and occupational, and metabolic risks or clusters of risks for 195 countries and territories, 1990–2017: a systematic analysis for the Global Burden of Disease Study 2017. Lancet. 2018;392(10159):1923‐1994. 30496105 10.1016/S0140-6736(18)32225-6 PMC6227755

[18] Roth GA , Mensah GA , Johnson CO , et al. Global burden of cardiovascular diseases and risk factors, 1990–2019. J Am Coll Cardiol. 2020;76(25):2982‐3021. 33309175 10.1016/j.jacc.2020.11.010 PMC7755038

[19] Neumann FJ , Sousa‐Uva M , Ahlsson A , et al. 2018 ESC/EACTS guidelines on myocardial revascularization. Eur Heart J. 2019;40(2):87‐165. 30615155 10.1093/eurheartj/ehy855

[20] Mejia OAV , Borgomoni GB , Lasta N , et al. Safe and effective protocol for discharge 3 days after cardiac surgery. Sci Rep. 2021;11(1):8979. 33903717 10.1038/s41598-021-88582-0 PMC8076282

[21] Ye Z , Chen J , Shen T , et al. Enhanced recovery after surgery (ERAS) might be a standard care in radical prostatectomy: a systematic review and meta‐analysis. Ann Palliat Med. 2020;9(3):746‐758. 32389010 10.21037/apm.2020.04.03

[22] Zhao Y , Zhang S , Liu B , Li J , Hong H . Clinical efficacy of enhanced recovery after surgery (ERAS) program in patients undergoing radical prostatectomy: a systematic review and meta‐analysis. World J Surg Oncol. 2020;18(1):131. 32552894 10.1186/s12957-020-01897-6 PMC7301489

[23] Huang ZD , Gu HY , Zhu J , et al. The application of enhanced recovery after surgery for upper gastrointestinal surgery: meta‐analysis. BMC Surg. 2020;20(1):3. 31900149 10.1186/s12893-019-0669-3 PMC6942370

[24] Moher D , Shamseer L , Clarke M , et al. Preferred reporting items for systematic review and meta‐analysis protocols (PRISMA‐P) 2015 statement. Syst Rev. 2015;4:1. 25554246 10.1186/2046-4053-4-1 PMC4320440

[25] Michaelis R , Tang V , Wagner JL , et al. Cochrane systematic review and meta‐analysis of the impact of psychological treatments for people with epilepsy on health‐related quality of life. Epilepsia. 2018;59(2):315‐332. 29313968 10.1111/epi.13989

[26] Shan C , Qiaogui W , Xueyun L , et al. Study on the application effect of enhanced recovery after surgery concept in pediatric cardiac surgery nursing. Chin Commun Doct. 2017;33(33):160‐161.

[27] Liping C . Analysis of application of enhanced recovery after surgery concept in cardiac surgery nursing. J Hunan Univ Tradit Chin Med. 2018;38(A01):756.

[28] Lan D . The clinical value of enhanced recovery after surgery concept guidance for cardiac surgery patients. World Latest Med Inf Dig. 2018;18(48):246‐247.

[29] Qilian G . The effect of enhanced recovery after surgery nursing on recovery and nursing satisfaction of surgical patients. World Latest Med Inf Dig. 2018;18(52):168+83.

[30] Junxiao G . Application of enhanced recovery after surgery concept in pediatric cardiac surgery nursing. China Rural Health. 2016;9(16):57.

[31] Zhengkun H , Sumi Z . Nursing effect analysis of enhanced recovery after surgery concept in patients after cardiac surgery. Med Forum. 2018;22(03):383‐384.

[32] Yan H . Clinical efficacy evaluation of enhanced recovery after surgery concept in cardiac surgery nursing. Int J Nurs. 2019;1(2):30.

[33] Renli H . Application of enhanced recovery after surgery concept in cardiac surgery nursing. Med Inf. 2020;33(z1):245‐246.

[34] Lijie J . Observation on the application of enhanced recovery after surgery concept in cardiac surgery nursing. Mod Dig Interv. 2019;24(A02):2263‐2264.

[35] Yanan L . Effect observation of FTS concept in pediatric cardiac surgery nursing. Clin Med Eng. 2018;25(11):1553‐1554.

[36] Yao L . Application analysis of enhanced recovery after surgery concept in cardiac surgery nursing. World Latest Med Inf Dig. 2016;16(80):374.

[37] Jing M , Weihua Z , Lirong Z , et al. Analysis of the application of enhanced recovery after surgery concept in cardiac surgery nursing. J Math Med. 2018;31(05):763‐765.

[38] Huaqin M . The application value of enhanced recovery after surgery concept in pediatric cardiac surgery nursing. Electr J Clin Med Lit. 2020;7(25):81+3.

[39] Yue P . The value evaluation of enhanced recovery after surgery concept in cardiac surgery nursing. Electr J Pract Clin Nurs Sci. 2018;3(48):16+8.

[40] Hua Q . Application of the concept of enhanced recovery after surgery in the nursing of cardiac surgery. Med Diet Health. 2019;17(5):1.

[41] Huan R . The application effect of enhanced recovery after surgery concept in cardiac surgery nursing. World Latest Med Inf Dig. 2019;19(71):355.

[42] Gaoqian S , Miaorui L . The effect of enhanced recovery after surgery concept on heart rate, plasma dosage and nursing satisfaction in cardiac surgery patients. Chin Rem Clin. 2019;19(17):3057‐3059.

[43] Hong T . Satisfaction analysis of enhanced recovery after surgery concept in cardiac surgery nursing. Chin Rem Clin. 2020;20(06):1026‐1027.

[44] Yanyan W . Application analysis of enhanced recovery after surgery concept in cardiac surgery nursing. Chin Commun Doct. 2018;34(20):162+4.

[45] Yan W . Application analysis of enhanced recovery after surgery concept in cardiac surgery nursing. Heilongjiang J Tradit Chin Med. 2020;49(05):268‐269.

[46] Ying W . Application of enhanced recovery after surgery concept in cardiac surgery nursing. China Pract Med. 2021;16(01):167‐169.

[47] Qiong W , Yanrong Z . The development and effect of enhanced recovery after surgery concept in cardiac surgery nursing. Electr J Pract Clin Nurs Sci. 2018;3(41):131‐132.

[48] Guiying X . The application of the concept of enhanced recovery after surgery in the nursing of cardiac surgery. Contemporary Nurses: Academic Edition (Midmonth Edition). 2018;25(6):35.

[49] Xiaohong Y , Xiaoyun L , Na L . The application value of enhanced recovery after surgery concept in pediatric cardiac surgery nursing. Mod J Integr Tradit Chin West Med. 2018;27(01):99‐102.

[50] Tiantian Z . Nursing effect of enhanced recovery after surgery concept combined with routine nursing on cardiac surgery patients. Med Diet Health. 2020;18(01):154+6.

[51] Dongmei Z . Nursing effect evaluation of fast rehabilitation nursing concept applied to cardiac surgery. Cardiovascular Disease Journal of Integrated Traditional Chinese and Western Medicine (Electronic Edition). 2016;4(30):111.

[52] Yan Z . Application of enhanced recovery after surgery concept in pediatric cardiac surgery nursing. Chin Manip Rehabil Med. 2016;7(04):74‐75.

[53] Lau CSM , Chamberlain RS . Enhanced recovery after surgery programs improve patient outcomes and recovery: a meta‐analysis. World J Surg. 2017;41(4):899‐913. 27822725 10.1007/s00268-016-3807-4

[54] Greer NL , Gunnar WP , Dahm P , et al. Enhanced recovery protocols for adults undergoing colorectal surgery: a systematic review and meta‐analysis. Dis Colon Rectum. 2018;61(9):1108‐1118. 30086061 10.1097/DCR.0000000000001160

[55] Li M , Zhang J , Gan TJ , et al. Enhanced recovery after surgery pathway for patients undergoing cardiac surgery: a randomized clinical trial. Eur J Cardiothorac Surg. 2018;54(3):491‐497. 29514224 10.1093/ejcts/ezy100

[56] Petersen J , Kloth B , Konertz J , et al. Economic impact of enhanced recovery after surgery protocol in minimally invasive cardiac surgery. BMC Health Serv Res. 2021;21(1):254. 33743698 10.1186/s12913-021-06218-5 PMC7981978

